# Looking at non-communicable diseases in Uganda through a local lens: an analysis using locally derived data

**DOI:** 10.1186/s12992-014-0077-5

**Published:** 2014-11-19

**Authors:** Jeremy I Schwartz, David Guwatudde, Rachel Nugent, Charles Mondo Kiiza

**Affiliations:** Department of Internal Medicine, Yale School of Medicine, New Haven, CT USA; Young Professionals Chronic Disease Network, Boston, MA USA; Uganda Initiative for Integrated Management of Non-communicable Diseases, Kampala, Uganda; Department of Epidemiology and Biostatistics, Makerere University School of Public Health, Kampala, Uganda; Department of Global Health, University of Washington, Seattle, WA USA; Department of Medicine, Mulago National Referral Hospital, Kampala, Uganda

**Keywords:** Non-communicable diseases, Chronic conditions, Low- and middle-income countries, Uganda, Health system financing, Multi-sector collaboration

## Abstract

The demographic and nutritional transitions taking place in Uganda, just as in other low- and middle-income countries (LMIC), are leading to accelerating growth of chronic, non-communicable diseases (NCDs). Though still sparse, locally derived data on NCDs in Uganda has increased greatly over the past five years and will soon be bolstered by the first nationally representative data set on NCDs. Using these available local data, we describe the landscape of the globally recognized major NCDs- cardiovascular disease, diabetes, cancer, and chronic respiratory disease- and closely examine what is known about other locally important chronic conditions. For example, mental health disorders, spawned by an extended civil war, and highly prevalent NCD risk factors such as excessive alcohol intake and road traffic accidents, warrant special attention in Uganda. Additionally, we explore public sector capacity to tackle NCDs, including Ministry of Health NCD financing and health facility and healthcare worker preparedness. Finally, we describe a number of promising initiatives that are addressing the Ugandan NCD epidemic. These include multi-sector partnerships focused on capacity building and health systems strengthening; a model civil society collaboration leading a regional coalition; and a novel alliance of parliamentarians lobbying for NCD policy. Lessons learned from the ongoing Ugandan experience will inform other LMIC, especially in sub-Saharan Africa, as they restructure their health systems to address the growing NCD epidemic.

## Introduction

*“In the African population of Uganda coronary heart disease is almost non-existent.”*

Thus opened Shaper and Jones’ seminal 1959 article in which they compared cholesterol levels in Asian and African communities of Kampala, Uganda’s capital city [1]. The former was experiencing high mortality rates from myocardial infarction while the latter was largely unaffected. At the time, this phenomenon was attributed to genetic or ethnic immunity [2]. The authors identified for the first time that a high-fat and low-fiber diet was associated with cardiovascular disease (CVD). They also wisely suggested an association of other lifestyle conditions such as physical inactivity with CVD. Now, 55 years later, the link between the major non-communicable diseases (NCDs)- CVD, diabetes mellitus (DM), cancer, and chronic respiratory disease (CRD)- and shared risk factors such as tobacco use, physical inactivity, unhealthy diet, and harmful use of alcohol is firmly established.

**Figure 1:**
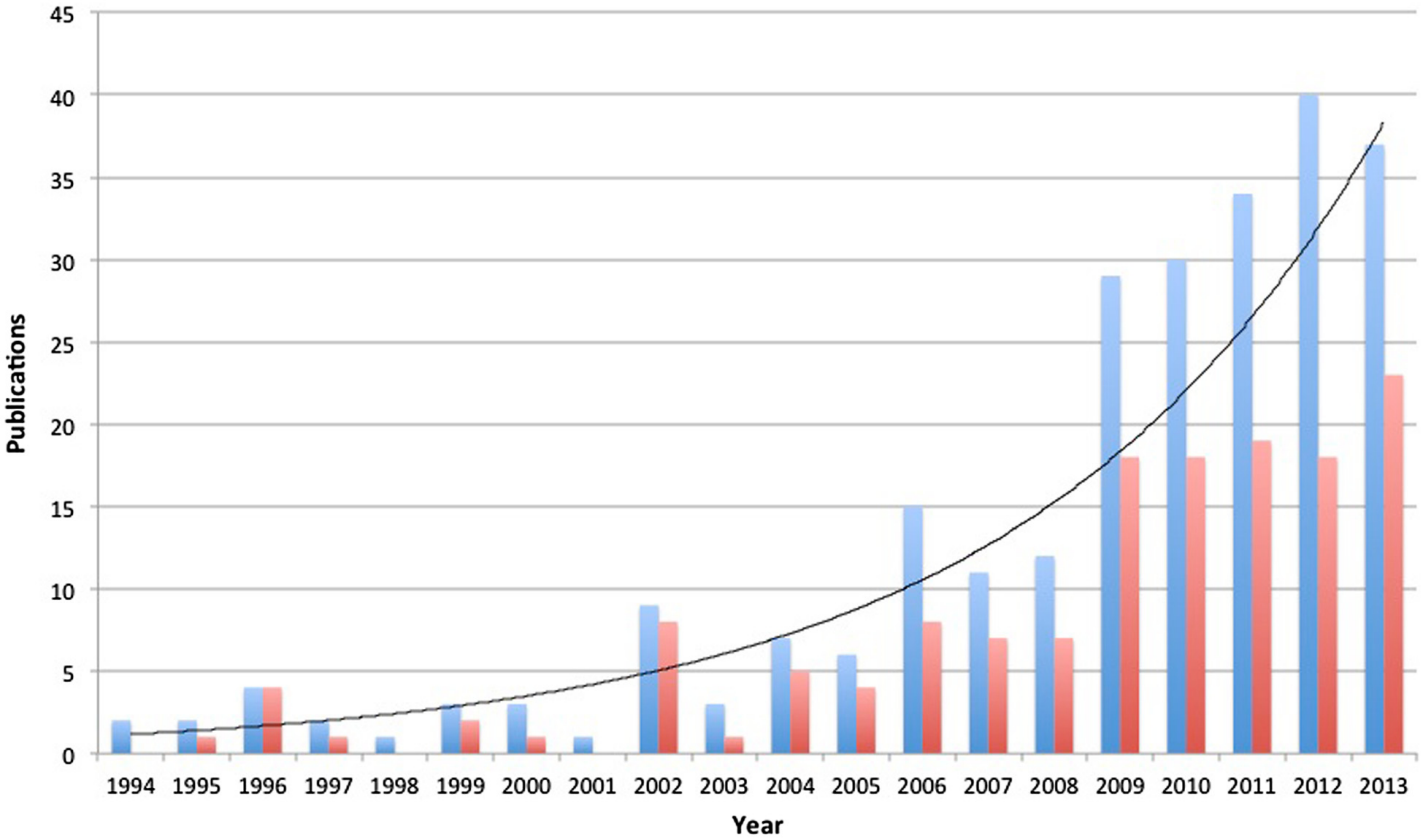
**Ugandan NCD publications, 1994–2013, (blue bars and trend line), and Ugandan affiliation of first or last author (red bars).**

The above example from Shaper and Jones illustrates the importance of locally generated data regarding risk factors, incident and prevalent disease, and morbidity and mortality. Unfortunately, such data are lacking in most LMIC, including Uganda. This poses a great challenge to effective health sector planning and resource allocation. In Uganda, over the last five years, there has been a marked increase in locally derived data about NCDs. Importantly, local authorship of this research has risen as well, from 40% between 1994–2003 to 60% between 2004–2013 (Figure 1). Herein we briefly review this literature with a preference toward population-based data, giving attention to first-of-their-kind studies for LMIC or sub-Saharan Africa (SSA). We focus on features unique to Uganda, discuss intervention costs, describe various strategies addressing NCDs, and offer recommendations for the future.

## Review

### Methods

Using OvidSP, we searched Medline, Global Health, and African Index Medicus databases for relevant English language literature. Search terms included a combination of: “Uganda”; “noncommunicable disease”; “non-communicable disease”; “chronic disease”; “diabetes mellitus”; “diabetes”; “cardiovascular disease”; “heart disease”; “respiratory disease”; “hypertension”; “cancer”; “hyperglycemia”; “kidney”; “renal”; “liver”; “asthma”; “injury”; “injuries”; “trauma”; “mental health”; “depression”; “tobacco”; “smoking”; “alcohol”; “diet”; “nutrition”; “physical activity”; “obesity”; “overweight”; “environmental”; “urbanization”. We supplemented electronic searches by hand searching reference lists and using resources from World Health Organization and the Global Burden of Disease Study 2010. Due to the limited number of papers, we considered all types of study designs, interventions, participants, and outcome measures. We excluded any papers published before 1980.

### A nation in transition

Like in many LMIC, the growing burden of NCDs in Uganda is part of an epidemiologic shift catalyzed by demographic and nutritional transitions. The population, currently 34 million, is growing more than 3 percent annually, among the highest on earth. At current rates, the population will double by 2030. The urban population surged from 800,000 in 1980 to 6.4 million in 2013 [3]. Urbanization is tightly linked with the nutritional transition, as prepackaged food consumption is higher among urban dwellers than rural [4].

### The current state of NCDs in Uganda

Aside from the four major NCDs, government policy includes other priority NCD areas including sickle cell disease, injury and disability, gender-based violence, mental health and substance abuse, integrated essential clinical care, oral health, and palliative care [5]. However, little data exist on some of these priority areas. Following years of delay, the first World Health Organization (WHO) Stepwise approach to Surveillance (STEPS) survey is underway in Uganda. STEPS, the standard instrument for collection, analysis, and dissemination of NCD data, will soon provide the first nationally representative information on NCDs.

#### Cardiovascular diseases and diabetes

**Figure 2:**
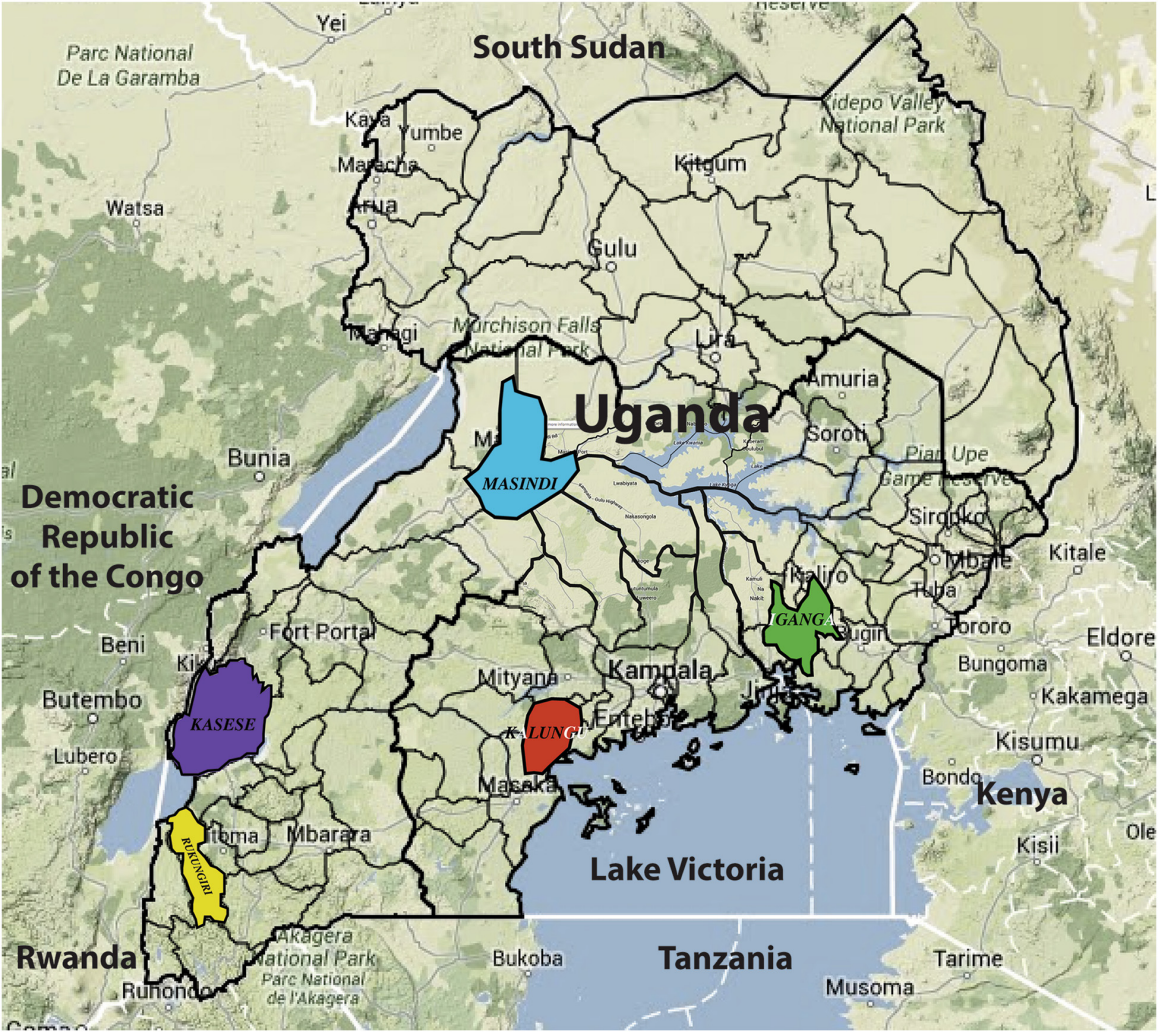
**Geographic distribution of population-based studies of diabetes, hypertension, stroke and NCD risk factors in Uganda.** Map is adapted from http://www.citypopulation.de/php/uganda-admin.php.

In the absence of national data, a number of small, geographically localized, population-based epidemiologic studies on hypertension (HTN), DM, stroke, and their risk factors have been conducted in Uganda. Though disease burden is substantial, our review of the literature does not suggest particular locally distinct characteristics or drivers. Thus, we have summarized the major findings of some of these studies in Table 1 and Figure 2 [6-11]. Rheumatic heart disease (RHD) remains the most common local cause of heart failure and a major cause of atrial fibrillation and stroke. The largest single-country childhood RHD prevalence study in SSA was conducted among randomly selected schoolchildren in Uganda in 2010. This study demonstrated the feasibility of school-based echocardiographic screening in this setting and revealed a prevalence approaching two percent [12].

**Table 1:** **Sample of population-based studies of diabetes, hypertension, stroke and associated risk factors in Uganda**

**Author/Year**	**District (color)**	**Study population**	**Key findings with prevalence**
**Mayega 2012** [6]	Iganga (green)	Cohort study; *Iganga-Mayuge Health and Demographic Surveillance Site;*Peri-urban and rural	-Overweight: 7.5% (males), 16.9% (females)
**Mayega 2014** [7]			
			-HBP: 20.5%
			-DM by hemoglobin a1c: 11.2%
			-DM by FPG: 4.8%
**Maher 2011** [8]	Kalungu (red)	Cohort study; *General Population Cohort;* Rural	-Overweight: 3.6% (males), 14.5% (females)
			-HBP: 22.5%
			-Probable DM by RPG: 0.4%
**Mondo 2013** [9]	Kasese (purple)	Cross-sectional study; Peri-urban and rural	-Overweight: 14.7% (males), 16.7% (females)
			-HBP: 21%
			-Physical inactivity: 51%
			-Daily smokers: 9.6%
**Wamala 2009** [10]	Rukungiri (yellow)	Cross-sectional study; Urban and rural	-HBP: 34%
			-Risk factors found to be independently associated with HBP: alcohol use, level of education, BMI
**Nakibuuka 2014** [11]	Wakiso (blue)	Cross-sectional study; Urban and rural	*Qualitative arm; quantitative results yet to be published*
			-Did not recognize brain as affected organ: 77%
			-Had no knowledge of stroke risk factors: 73%
			-Would go to hospital if stroke symptoms: 85%

HBP high blood pressure, DM diabetes mellitus, FPG fasting plasma glucose, BMI body mass index, RPG random plasma glucose.

#### Cancers

**Figure 3:**
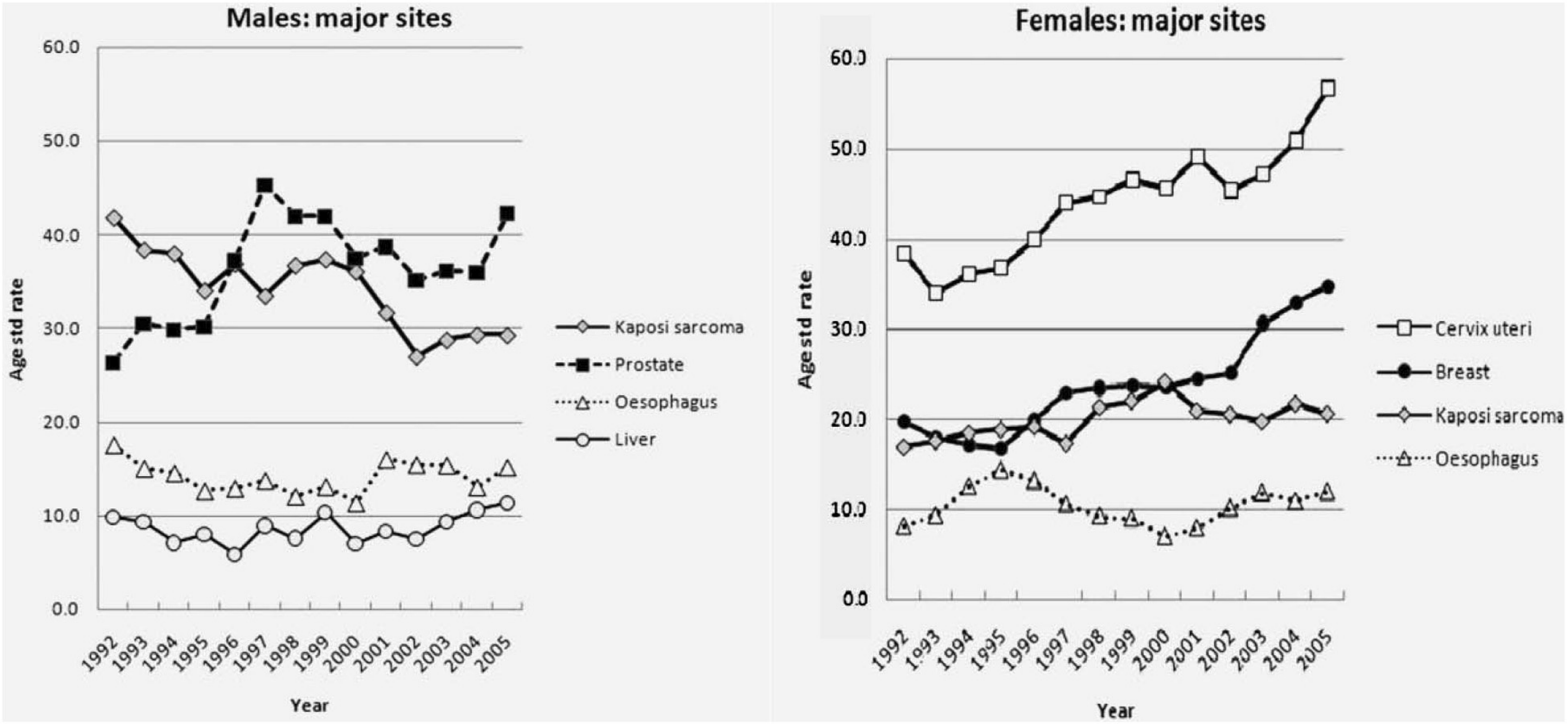
**Trends in age-standardized incidence rates of selected cancers in males and females in Kampala, Uganda, 1991–2006.** (Adapted from Parkin [14]).

The epidemiology of cancer in Uganda has shifted greatly since Dr. Burkitt embarked on his “tumour safari” around East and Central Africa in the early 1960s and correctly hypothesized that the distinctive pediatric lymphoma of the jaw, now bearing his name, was virally-mediated [13]. Cancer epidemiology came to mirror the HIV epidemic. Though AIDS-associated malignancies such as epidemic Kaposi sarcoma (KS) overshadowed the formerly prevalent endemic KS, these have now declined. The past two decades have seen a steady increase in cancers typically associated with “Western lifestyles”, particularly those of the breast and prostate (Figure 3) [14].

#### Chronic respiratory diseases

Discussion of CRD in Uganda demands attention to both tobacco use and indoor air pollution, as illustrated by a mixed-methods study in rural Masindi. Most of the 16% of adults diagnosed with chronic obstructive pulmonary disease were women. Though tobacco use was more common in men, daily exposure to poorly ventilated indoor smoke from biomass fuels was significantly higher in women, who are expected to cook despite chronic cough. While in non-tobacco-growing areas chronic cough was associated with tuberculosis and was highly stigmatized, it was considered part of everyday life and not considered a health problem in tobacco-growing areas [15].

#### Mental illness

The 20-year-long civil war that raged in the North displaced millions, violated the peace of a generation, and witnessed the recruitment of 25,000 children as soldiers into the Lord’s Resistance Army [16]. The mental health literature, therefore, largely emanates from the North and focuses on the sequelae of war among the region’s youth. One representative study illustrated the disturbing prevalence of post-traumatic stress disorder and behavioral problems among children abducted during the war [17]. The first community-based randomized control trial of psychotherapy for depression in a LMIC was conducted in Northern Uganda. This successful trial trained lay counselors to deliver group psychotherapy [18].

#### Risk factors

Uganda has among the highest alcohol consumption per capita and a low detection of problem drinking in the healthcare setting [19]. A study in the North showed a tight correlation between suicide and alcohol consumption while one in Kampala demonstrated high rates of childhood suicidal tendency associated with alcohol-related parental neglect [20,21]. Intimate partner violence (IPV) is also common in Uganda and is fueled by problem drinking, unlike in some neighboring countries with high IPV rates and low alcohol consumption. Nearly half of Ugandan women have experienced IPV and those whose partners drink frequently have a six-fold higher risk than those whose partners abstain from alcohol. The poorest and least educated women experience the highest rates of IPV [22].

Road traffic accidents (RTA) are an increasing problem on Uganda’s congested byways, with the second highest rate of RTA in SSA and among the highest associated fatality rate globally [23]. A pre-hospital trauma care program for lay first-responders in Kampala was shown to be feasible and cost-effective [24].

### NCD financing in Uganda

#### Analyzing the cost of care

Given the general lack of NCD cost analysis in SSA and LMICs, it is unsurprising that, to the best of the authors’ knowledge, there have not been any published studies of NCD-related costs in Uganda. An upcoming comprehensive review of global NCD cost publications from LMIC confirms that primary prevention of CVD, stroke, and diabetes is far less expensive and has lower unit costs than treatment interventions for those conditions. Further, outpatient treatment is far less costly than inpatient treatment, so it is critical to have an effective referral system that directs patients to the proper facility [25]. Using China, an upper-middle income country, as an example, population-level primary prevention of diabetes- through media campaigns- costs well under one USD per capita per year. However, treatment of uncomplicated diabetes costs nearly 1000USD and treatment of complicated diabetes costs nearly 2,500USD [26,27].

A costed NCD strategy is a starting place for determining the scope and pace of scaling up NCD services in Uganda. There are many existing tools and sources of costing information [28]. Although it will take time for Uganda to develop a full array of costs for providing NCD services, the success of an NCD strategy will ultimately depend upon a local understanding of costs and benefits.

#### Ministry of health financing for NCDs

In 2006, The Ugandan Ministry of Health (MoH) established the Programme for the Prevention and Control of NCDs. This small unit is one of nine divisions within the Department of Community Health and is responsible for all national NCD-related activities. A separate division, Health Promotion, is actually responsible for promoting healthy lifestyle changes in the population. According to the Government of Uganda Ministry of Health Ministerial Policy Statement 2014/2015, the NCD Programme is allocated 0.01 percent of the total MoH budget, representing three percent of the Departmental budget, or approximately 27,000USD per annum. The Programme budget is currently supplemented by a five-year grant from the World Diabetes Federation (WDF) that expires at the end of 2017 and that brings the total budget to approximately 270,000USD. For the current fiscal year, NCD Programme funding was increased to 25% of the total Departmental budget to allow for the completion of STEPS (Table 2) [unpublished data, Government of Uganda Ministry of Health].

**Table 2:** **Ministry of health financing for NCDs [provided by personal communication: Ministry of Health Ministerial Policy Statement 2014/2015, the Republic of Uganda]**

	**Approximate budget (USD), 2014-15**	**Percent of total MOH budget**
Ministry of Health	240,000,000	-
Source: Government of Uganda	20,000,000	8.3
Source: External financing	220,000,000	91.7
Department of Community Health	902,000	0.37
Programme for the Prevention and Control of NCDs	27,000^*^	0.011

^*^The NCD Programme budget was increased from 3% (27,000USD) of Department of Community Health budget to 25% (250,000USD) for 2014–15 to fund the STEPS survey.

### Current efforts to combat NCDs in Uganda

#### Public sector NCD capacity

An NCD needs assessment conducted in 2013 characterized, for the first time in Uganda, the capacity of regional and general hospitals and high-level (Level IV) health centers to provide NCD-related services. It evaluated human resources, equipment, drug supply, and laboratory capabilities in 54 centers. Despite variability, none of the facilities examined were found to meet WHO standards for essential tools and medicines needed to implement effective NCD interventions. All health facilities had experienced stock-outs within the last year of nearly all classes of hypertension and diabetes medications. Furthermore, the availability of NCD equipment and services was inadequate. Only 38% of electrocardiogram machines available in these facilities, for example, were functional (Table 3). This study also surveyed the attitude and confidence of HCWs regarding NCDs. While most medical officers and physicians (62.5% and 100%, respectively) sampled felt that their clinical training prepared them adequately to manage NCDs, only 25% of clinical officers agreed. Only 43% of medical officers and 44% of physicians felt that their facility had the capacity to treat NCDs. Confidence in screening for, and treating, common NCDs varied greatly by cadre of HCW (Table 4) [unpublished data, Rogers H. Assessment of the capacity of Ugandan health facilities, personnel, and resources to prevent and control noncommunicable diseases. Masters thesis. Yale School of Public Health. 2014.]

**Table 3:** **Availability of NCD services and equipment by health facility type (adapted from Rogers H 2014)**

	**Regional referral hospitals (n = 13)- No. (%)**	**General hospitals (n = 27)- No. (%)**	**Health center IV (n = 14)- No. (%)**
**Service**			
Body Mass Index	6 (46)	5 (19)	0 (0)
Blood pressure	13 (100)	24 (89)	12 (86)
Blood glucose	11 (85)	23 (85)	11 (79)
Urine analysis	9 (69)	22 (82)	12 (86)
Individual patient NCD education	9 (69)	17 (63)	5 (36)
Group NCD education	12 (92)	18 (67)	4 (29)
**Equipment**			
Stethoscope	8 (62)	16 (60)	7 (50)
Blood pressure cuff, standard size	4 (31)	9 (33)	8 (57)
Electrocardiogram- available/[functional]	3 (21)/ [1 (33)]	4 (15)/ [2 (50)]	1 (7)/ [0 (0)]

**Table 4:** **Confidence in NCD management by cadre of HCW- (adapted from Rogers H 2014)**

	**Nurse (n = 32)**		**Clinical officer (n = 16)**		**Medical officer (n-19)**		**Physician (n = 10)**	
	**Confident** ^*^ **-No. (%)**	**Not confident** ^**^**^ **- No. (%)**	**Confident- No. (%)**	**Not confident- No. (%)**	**Confident- No. (%)**	**Not confident- No. (%)**	**Confident- No. (%)**	**Not confident- No. (%)**
Hypertension treatment	14 (44)	18 (56)	12 (75)	4 (25)	19 (100)	0 (0)	10 (100)	0 (0)
Diabetes treatment	14 (44)	18 (56)	4 (44)	9 (56)	17 (90)	2 (10)	10 (100)	0 (0)
Asthma treatment	21 (66)	11 (34)	14 (88)	2 (12)	18 (95)	1 (5)	10 (100)	0 (0)
Cervical cancer screening	7 (22)	25 (78)	0 (0)	16 (100)	8 (42)	11 (58)	6 (60)	4 (40)
Depression screening/treatment	10 (31)	22 (69)	6 (38)	9 (62)	10 (53)	9 (47)	incomplete	
Tobacco abuse treatment	11 (34)	21 (66)	3 (19)	13 (81)	4 (21)	15 (79)	incomplete	
Alcohol abuse treatment	17 (53)	15 (47)	4 (25)	12 (75)	6 (32)	13 (68)	Incomplete	

^*^“Confident” combines “very confident” and “confident” in the original survey.

^^^“Not confident” combines “somewhat” and “not at all” in the original survey.

#### NCD-focused collaborations

The heterogeneity of NCDs and the strong influence of social determinants of health on risk factor acquisition means that traditional vertical models ineffectively address NCDs. Instead, Uganda must focus on multi-sector efforts and a diagonal approach to health system strengthening, moving from disease-specific endeavors towards integrated health delivery platforms [29]. A number of local efforts toward that end are underway.

The Uganda Initiative for Integrated Management of NCDs (UINCD) is addressing the fragmented management of NCDs and inadequate NCD-related training of healthcare workers by developing and studying models of integrated care delivery and education [30]. UINCD has received administrative and start-up financial support from the Yale Global Health Leadership Institute and unites MoH, academia, and health center leadership in collaboration with researchers from Yale University. UINCD aims to scale up integration efforts nationwide and partner with HIV organizations to leverage existing infrastructure such as training models, pharmaceutical supply chains, laboratory capabilities, and clinical care. Another multidisciplinary collaboration, Global Partners in Anesthesia and Surgery (GPAS), focuses on capacity building within the surgical workforce and implementation of strategies to improve surgical care [31]. Surgical issues, often omitted from discussion of NCDs, demand attention specifically related to diabetic complications and road traffic injuries, both of which GPAS addresses. GPAS is supported by private foundation support and small academic grants.

The Uganda NCD Alliance (UNCDA) is working to build a strong civil society coalescing around NCDs. Supported by the Danish Civil Society Fund and guided by academics and the global NCD Alliance, UNCDA is host to the East Africa NCD Alliance Initiative. This is an ambitious South-South collaborative uniting the nascent NCD Alliances in each country in the region and addressing the inclusion of NCDs in the post-2015 development agenda. The East Africa NCD Alliance Initiative began work in early 2014 and has already had some major successes. Using the global NCD Alliance Advocacy Toolkit, the Initiative conducted a benchmarking survey to document an evidence-based account of the NCD response in East Africa from a civil society perspective [32,33]. A few highlights from the report of this survey include: 1. All countries in the region are in the process of formulating national NCD plans in concert with civil society organizations. However, only Kenya has developed and endorsed national NCD targets; 2. NCDs are prioritized in national health and development plans, though implementation remains weak and the resource allocation has been insufficient; and 3. Although surveillance and monitoring of NCDs in East Africa has improved, NCDs are not sufficiently integrated into national health information management systems and health personnel are limited in their capacity for NCD surveillance and data collection. This Report was then used to inform the writing of The East Africa Civil Society Charter, a call to action for regional governments, institutions, and the global community. The Charter was signed by regional stakeholders in Entebbe, Uganda in June 2014 [34]. These activities were then presented at United Nations High Level Review on NCDs in July 2014 [35]. UNCDA continues to work locally through regional branches that provide education and support to patients, families, and communities and also nationally through advocacy campaigns and lobbying efforts.

An influential group unique, to the authors’ knowledge, within SSA is the Uganda Parliamentary Forum on NCDs. This group of lawmakers advocates within Parliament for the advancement of the NCD agenda. The Forum has been most active addressing the WHO Framework Convention on Tobacco Control, which, though signed in Uganda in 2004 and ratified in 2007, still lingers in Parliament. The Forum has organized the Ministries of Trade, Agriculture, and Tourism in a nationwide public awareness and advocacy campaign around tobacco control [36].

## Discussion

This review relies on currently available, locally derived data. The generalizability of these data is limited by the small sample sizes and geographic localization of the respective studies. However, these studies offer a local perspective that is largely lacking from, or sometimes disagrees with, the data available from large-scale, global analyses. A few such examples are worth noting. First, the WHO Global Health Observatory Data Repository suggests that Uganda’s population growth rate is −3.4%, in contrast to the +3% rate suggested by local data. At the same time, the estimate of the urban dwellers (16%) approximates and that of the local data (18.8%) [37]. Second, the reported national prevalence of hypertension in the World Health Organization’s Uganda NCD Country Profile is 34% [38]. This represents the upper range of the local data presented herein; data that is more likely to capture the expected prevalence differences between urban and rural settings. Third, some important data do not exist in the global databases. For instance, by looking at the WHO Global Health Expenditure Database, one would be unable to estimate the Ugandan MoH financing of its NCD Programme [39]. As would be suggested by the local data, GBD 2010 estimates that a number of the NCDs and risk factors described herein (including IPV, RTA, depression, stroke, and CRD) are among the leading causes of disability-adjusted life years (DALYs) in Uganda. Between 1990 and 2010, RTA and IPV increased more than any other causes of DALYs in Uganda [40].

Though nationally representative data from STEPS should become available shortly, this survey is fairly narrow in scope. STEPS has three sections: Risk factors (tobacco and alcohol use, fruit and vegetable consumption, and physical activity); Physical measurements (body mass index, waist circumference, and blood pressure); and Biochemical measurements (blood glucose and cholesterol). STEPS will not provide nationally representative data on IPV, RTA, mental illness, or biomass fuel exposure, among others. Therefore, the research highlighted in this review serves as a critically important complement to the type of information that we will learn from Uganda’s STEPS survey. The fact that local researchers are conducting an increasing percentage of this work speaks to a growing academic capacity in Uganda that must be embraced and nurtured.

The availability of local data is essential for the formation of policy that addresses NCDs and the health system restructuring that will enable support of chronic disease management. However, such data alone will be insufficient to move policy into implementation. There are numerous obstacles that stand between good research, well written policy, and effectively implemented programs. These can include government bureaucracy, health sector financing, and health system capacity to effect change [41]. However, we can learn from successful examples such as that of behavior change messaging for HIV control early in the Ugandan epidemic. An environment existed that embraced evidence-based health messaging. A government and bureaucracy that engaged actively with donors and non-governmental organizations enabled such an environment. This, in part, is credited with the dramatically decrease HIV prevalence in Uganda in the 1990s [42].

## Conclusion

Today in Uganda, several challenges must be addressed to ensure that the relatively new surge in local NCD-related research and a rich environment of cross-sector collaborations translate into effective policy and implementation. First, funding for NCDs is grossly inadequate. Along with greater understanding of risk factor and disease epidemiology, a focus on NCD costing will be critical if NCD financing is to attain a level proportional to the burden. The sustainability of the 92% of MoH’s budget coming from external sources depends upon the international political environment and the consistency of donor support. The 90% of current NCD Programme funding supplied by WDF, too, is unsustainable. Second, the failure of most high-level health facilities to meet minimum standards for NCD interventions is concerning. As integrated models for prevention and care are evaluated, attention must be given to cost-effectiveness and leveraging available resources. Finally, stronger governance and a robust civil society are needed in order to catalyze the implementation of policies such as tobacco control and to counter the increasing pressures of transnational corporations. The success of the NCD agenda will rely on the cooperation and fortitude of multiple sectors as well as a strengthening civil society that can shed light on the social injustices and socioeconomic determinants of health that help drive the Ugandan NCD epidemic.

## Abbreviations

BMI: Body mass index
COPD: Chronic obstructive pulmonary disease
CRD: Chronic respiratory disease
CVD: Cardiovascular disease
DALY: Disability-adjusted life year
DM: Diabetes mellitus
FPG: Fasting plasma glucose
GBD: Global Burden of Disease
GPAS: Global Partners in Anesthesia and Surgery
HBP: High blood pressure
HTN: Hypertension
IPV: Intimate partner violence
KS: Kaposi sarcoma
LMIC: Low- and middle-income country
MoH: Ministry of Health
NCD: Non-communicable disease
RPG: Random plasma glucose
RHD: Rheumatic heart disease
RTA: Road traffic accidents
SSA: Sub-Saharan Africa
STEPS: Stepwise approach to Surveillance
UINCD: Uganda Initiative for Integrated Management of Non-Communicable Diseases
UNCDA: Uganda Non-Communicable Disease Alliance
UNMHCP: Uganda National Minimum Health Care Package
WDF: World Diabetes Federation
WHO: World Health Organization

## References

[1] Shaper AG, Jones KW (2012). Serum-cholesterol, diet, and coronary heart-disease in Africans and Asians in Uganda: 1959. Int J Epidemiol.

[2] Mayosi BM, Forrester T (2012). Commentary: ‘serum-cholesterol, diet, and coronary heart-disease in Africans and Asians in Uganda’ by AG Shaper and KW Jones. Int J Epidemiol.

[3] **Uganda Bureau of Statistics Statistical Abstract 2013.** [http://www.ubos.org/onlinefiles/uploads/ubos/pdf%20documents/abstracts/Statistical%20Abstract%202013.pdf]

[4] Raschke V, Cheema B (2007). Colonisation, the New World Order, and the eradication of traditional food habits in East Africa: historical perspective on the nutrition transition. Public Health Nutr.

[5] **Government of Uganda Ministry of Health- Health sector strategic & investment plan: Promoting people’s health to enhance socio-economic development.** [http://www.unicef.org/uganda/HSSIP_Final.pdf]

[6] Mayega RW, Makumbi F, Rutebemberwa E, Peterson S, Ostenson CG, Tomson G, Guwatudde D (2012). Modifiable socio-behavioural factors associated with overweight and hypertension among persons aged 35 to 60 years in eastern Uganda. PLoS One.

[7] Mayega RW, Guwatudde D, Makumbi FE, Nakwagala FN, Peterson S, Tomson G, Ostenson CG (2014). Comparison of fasting plasma glucose and haemoglobin A1c point-of-care tests in screening for diabetes and abnormal glucose regulation in a rural low income setting. Diabetes Res Clin Pract.

[8] Maher D, Waswa L, Baisley K, Karabarinde A, Unwin N, Grosskurth H (2011). Distribution of hyperglycaemia and related cardiovascular disease risk factors in low-income countries: a cross-sectional population-based survey in rural Uganda. Int J Epidemiol.

[9] Mondo CK, Otim MA, Akol G, Musoke R, Orem J (2013). The prevalence and distribution of non-communicable diseases and their risk factors in Kasese district, Uganda. Cardiovasc J Afr.

[10] Wamala J, Karyabakabo Z, Ndungutse D, Guwatudde D (2009). Prevalence factors associated with hypertension in Rukungiri District, Uganda - A community-based study. Afr Health Sci.

[11] Nakibuuka J, Sajatovic M, Katabira E, Ddumba E, Byakika-Tusiime J, Furlan AJ (2014). Knowledge and Perception of Stroke: A Population-Based Survey in Uganda.

[12] Beaton A, Okello E, Lwabi P, Mondo C, McCarter R, Sable C (2012). Echocardiography screening for rheumatic heart disease in Ugandan schoolchildren. Circulation.

[13] Burkitt D (1962). A “tumour safari” in East and Central Africa. Br J Cancer.

[14] Parkin DM, Nambooze S, Wabwire-Mangen F, Wabinga HR (2010). Changing cancer incidence in Kampala, Uganda, 1991–2006. Int J Cancer.

[15] van Gemert F, Chavannes N, Nabadda N, Luzige S, Kirenga B, Eggermont C, de Jong C, van der Molen T (2013). Impact of chronic respiratory symptoms in a rural area of sub-Saharan Africa: an in-depth qualitative study in the Masindi district of Uganda. Prim Care Respir J.

[16] Klasen F, Oettingen G, Daniels J, Adam H (2010). Multiple trauma and mental health in former Ugandan child soldiers. J Trauma Stress.

[17] McMullen JD, O’Callaghan PS, Richards JA, Eakin JG, Rafferty H (2012). Screening for traumatic exposure and psychological distress among war-affected adolescents in post-conflict northern Uganda. Soc Psychiatry Psychiatr Epidemiol.

[18] Bass J, Neugebauer R, Clougherty KF, Verdeli H, Wickramaratne P, Ndogoni L, Speelman L, Weissman M, Bolton P (2006). Group interpersonal psychotherapy for depression in rural Uganda: 6-month outcomes: randomised controlled trial. Br J Psychiatry.

[19] Kullgren G, Alibusa S, Birabwa-Oketcho H (2009). Problem drinking among patients attending primary healthcare units in Kampala, Uganda. Afr J Psychiatry.

[20] Freers J, Mayanja-Kizza H, Ziegler JL, Rutakingirwa M (1996). Echocardiographic diagnosis of heart disease in Uganda. Trop Doct.

[21] Swahn MH, Palmier JB, Kasirye R, Yao H (2012). Correlates of suicide ideation and attempt among youth living in the slums of Kampala. Int J Environ Res Public Health.

[22] Tumwesigye NM, Kyomuhendo GB, Greenfield TK, Wanyenze RK (2012). Problem drinking and physical intimate partner violence against women: evidence from a national survey in Uganda. BMC Public Health.

[23] **Uganda Road Sector Support Initiative.** [http://www.ugandaroadsector.org/RoadSafety.php]

[24] Jayaraman S, Mabweijano JR, Lipnick MS, Caldwell N, Miyamoto J, Wangoda R, Mijumbi C, Hsia R, Dicker R, Ozgediz D (2009). First things first: effectiveness and scalability of a basic prehospital trauma care program for lay first-responders in Kampala, Uganda. PLoS One.

[25] Brouwer E, Watkins D, Olson Z, Goett J, Levin C: **Health system and provider costs for prevention and treatment of cardiovascular and related conditions in low and middle-income countries: a systematic review.** In *DCP3 Working Paper.* Seattle: University of Washington Department of Global Health; 2014. Available at www.dcp-3.org. 10.1186/s12889-015-2538-z PMC4660724 26612044

[26] Zhang YL, Gao WG, Pang ZC, Sun JP, Wang SJ, Ning F, Song X, Kapur A, Qiao Q (2012). Diabetes self-risk assessment questionnaires coupled with a multimedia health promotion campaign are cheap and effective tools to increase public awareness of diabetes in a large Chinese population. Diab Med.

[27] Wang W, Fu CW, Pan CY, Chen W, Zhan S, Luan R, Tan A, Liu Z, Xu B (2009). How do type 2 diabetes mellitus-related chronic complications impact direct medical cost in four major cities of urban China?. Value Health.

[28] **Technical Review of Costing Tools for the Health MDGs: Final Report.** [http://www.who.int/pmnch/topics/economics/costoolsreviewpack.pdf]

[29] Binagwaho A, Muhimpundu MA, Bukhman G (2014). 80 under 40 by 2020: an equity agenda for NCDs and injuries. Lancet.

[30] **Uganda Initiative for Integrated Management of Non-Communicable Diseases.** [http://uincd.org]

[31] **Global Partners in Anesthesia and Surgery.** [http://globalsurgery.org]

[32] The NCD Alliance: **Non-Communicable Diseases: Join the Fight- An Online Advocacy Toolkit.** [http://ncdalliance.org/sites/default/files/rfiles/NCD%20Toolkit%20FINAL.pdf]

[33] East Africa NCD Alliance Initiative: **A Civil Society Benchmark Report: Responses to NCDs in East Africa.** [http://www.uncda.org/sites/default/files/resources/East%20Africa%20NCD%20Alliance%20Civil%20Society%20Survey%20Report_v5_layouted.pdf]

[34] East Africa NCD Alliance Initiative: **The East Africa Civil Society NCD Charter- From Committment to Action: Accelerating the NCD Response in East Africa.** [http://www.uncda.org/sites/default/files/resources/East%20Africa%20Civil%20Society%20NCDA%20Charter_final_layouted.pdf].

[35] Yonga G: **Statement at United Nations- Informal interactive hearing with non-governmental organizations, civil society organizations, the private sector and academia on the prevention and control of non-communicable diseases.** 2014 [http://papersmart.unmeetings.org/media2/3501293/statement-by-gerald-yonga.pdf]

[36] Ninsiima R, Namumbya R: **Draft law just made tobacco too expensive.** [http://www.observer.ug/index.php?option=com_content&task=view&id=23570&Itemid=114]

[37] **Global Health Observatory Data Repository.** [http://apps.who.int/gho/data/node.country.country-UGA?lang=en]. 10.1080/02763869.2019.1693231 32069199

[38] **World Health Organization - Noncommunicable Disease Country Profiles, 2014.** [http://www.who.int/nmh/countries/uga_en.pdf?ua=1]

[39] **Global Health Expenditure Database.** [http://apps.who.int/nha/database/Select/Indicators/en]

[40] **Global Burden of Disease Profile: Uganda.** [http://www.healthdata.org/sites/default/files/files/country_profiles/GBD/ihme_gbd_country_report_uganda.pdf]

[41] Philpott A, Maher D, Grosskurth H (2002). Translating HIV/AIDS research findings into policy: lessons from a case study of ‘the Mwanza trial’. Health Policy Plan.

[42] Parkhurst JO, Lush L (2004). The political environment of HIV: lessons from a comparison of Uganda and South Africa. Soc Sci Med.

